# Association between atopic disease and anemia in pediatrics: a cross-sectional study

**DOI:** 10.1186/s12887-019-1836-5

**Published:** 2019-11-25

**Authors:** Kiyon Rhew, Jung Mi Oh

**Affiliations:** 1 College of Pharmacy, Seoul National University, 1 Gwanak-ro, Gwanak-gu, Seoul, 08826 Republic of Korea; 2 College of Pharmacy, Dongduk Women’s University, 60 Hwarang-ro 13-gil, Seongbuk-gu, Seoul, 02748 Republic of Korea; 3 Research Institute of Pharmaceutical Sciences, Seoul National University, 1 Gwanak-ro, Gwanak-gu, Seoul, 08826 Republic of Korea

**Keywords:** Atopic disease, Anemia, Allergic rhinitis, Asthma, Atopic dermatitis

## Abstract

**Background:**

Atopic diseases, such as atopic dermatitis, allergic rhinitis, and asthma, are inflammatory diseases common in pediatric patients. This study investigated whether these inflammatory atopic diseases were associated with anemia in pediatrics.

**Methods:**

A cross-sectional study was conducted using a pediatric dataset from the Health Insurance Review and Assessment Service (HIRA) of South Korea in 2016. Multivariable logistic regression, adjusting for demographic covariates was used for analyse the association between atopic disease and iron deficiency anemia (IDA).

**Results:**

A total of 846,718 pediatric patients were included in the study. Of these, 19,594 (2.31%) had a diagnosis of IDA. The logistic regression analyses including covariates revealed there were association between atopic disease and IDA. The adjusted OR (aOR) of IDA was 1.42 (95% CI, 1.37–1.47) for atopic dermatitis, 1.25 (95% CI, 1.21–1.29) for allergic rhinitis, and 1.71 (95% CI, 1.65–1.76) for asthma. IDA was more prevalent in patients with multiple comorbid atopic diseases, with aOR of 1.30 (95% CI, 1.25–1.35), 1.81 (95% CI, 1.73–1.89), and 2.58 (95% CI, 2.43–2.73) for 1, 2, or 3 atopic diagnoses. There was no evidence of multicollinearity among covariates.

**Conclusions:**

Our findings suggest that atopic disease was associated with IDA. Further study is needed to clarify the distinction between IDA and/or AI to better understand the cause of anemia in patients with inflammatory diseases.

## Background

Atopic diseases, such as atopic dermatitis, allergic rhinitis, and asthma, are inflammatory diseases common in pediatrics [1, 2]. Atopic diseases are often comorbid with other diseases; indeed, the prevalence of infectious disease, autoimmune disease, and mental illness is higher in these patients than those without atopic disease [3–5], and these conditions, singly or in combination, can decrease patients’ quality of life and increase morbidity and mortality [6–8].

No study has definitively evaluated whether the persistent inflammatory state of atopic disease impacts the risk of developing anemia. Although one study reported an association between higher iron levels and a lower prevalence of asthma [9], the design was cross-sectional, making cause and effect difficult to discern. Another study showed that the prevalence of anemia in children with atopic disease is higher than in those without atopic disease [10], but these findings relied on caregiver questionnaires, and the effects of other diseases or drugs that simultaneously affect anemia and atopic disease were not partitioned.

Therefore, in this study, we investigated whether the chronic inflammatory state of atopic patients affects their risk for anemia while controlling for potentially confounding covariates. We considered the risk factors for both atopic disease and anemia.

## Methods

### Study population

South Korea has a universal health insurance system, into which approximately 98% of all citizens are enrolled. We used a dataset, Health Insurance Review and Assessment Service - Pediatric Patient Sample, (HIRA-PPS-2016), that randomly stratified 10% of the pediatric patients (~ 1,100,000) under 20 years of age who used a medical institution during the year 2016 [11]. HIRA reported that almost 90% of Koreans visit medical institutions at least once a year [11]. In South Korea, health care providers report diagnostic codes when prescribing a medication or procedure, and patients are required to physically visit their physician to receive prescriptions. Thus, the requirement of diagnostic codes and absence of refill and telemedicine models results in a dataset with no missing diagnostic codes in the claim data.

The inclusion criterion in this study was patient age under 18 years. Exclusion criteria for this study were: 1) patients who were diagnosed with an anemia other than IDA, 2) patients who were diagnosed with two or more types of anemia. We chose IDA to evaluate if inflammation of atopic diseases increases the prevalence of anemia since the hematologic characteristics of IDA is similar with anemia of inflammation (AI). In addition, other types of anemia except IDA, such as hemolytic anemia, aplastic anemia or folate deficiency anemia, were excluded in this study because of their low prevalence and different mechanisms of etiology. Diagnosis of AI was very low prevalence in children and used for patients with specific chronic condition such as CKD or cancer, so it was also excluded in this study.

### Definition of diseases

We included atopic dermatitis, allergic rhinitis, and asthma as atopic disease based on the presence of the diagnostic code corresponding to one or more of these diseases. Diagnostic codes for these diseases and covariate diseases (see below) are based on The Korean Standard Classification of Disease and Cause of Death-7 (KCD-7) was used for definition of disease (Additional file 1: Table S1). The KCD-7 reflects the update of the International Classification of Diseases 10th Revision (ICD-10) and refines the Korean subtypes of disease and rare diseases.

### Covariates

Covariates were selected based on patient characteristics and common risk factors for anemia and atopic disease. Patient characteristics (age, sex, and insurance type), medical conditions, and medication were included in our analyses as covariates. We included systemic infection, chronic kidney disease (CKD), mental disorder, chronic inflammation, and cancer as covariates for medical condition. Systemic steroids, cyclosporine, and methotrexate were included as covariates for medications. Specific diseases and corresponding KCD-7 codes are listed in Additional file 1: Table S1.

### Statistical analysis

We estimated crude odds ratio (cOR) and adjusted odds ratio (aOR) with 95% confidence interval (CI). We utilized stepwise multivariate binary logistic regression analysis, including the covariates described above. Multicollinearity test were performed since we included quite a few variables as covariates. All statistical analyses were performed in SAS 9.4 (SAS Institute Inc., Cary, NC, USA), and results were considered statistically significant if the *P*-value was less than 0.05.

## Results

**Fig. 1:**
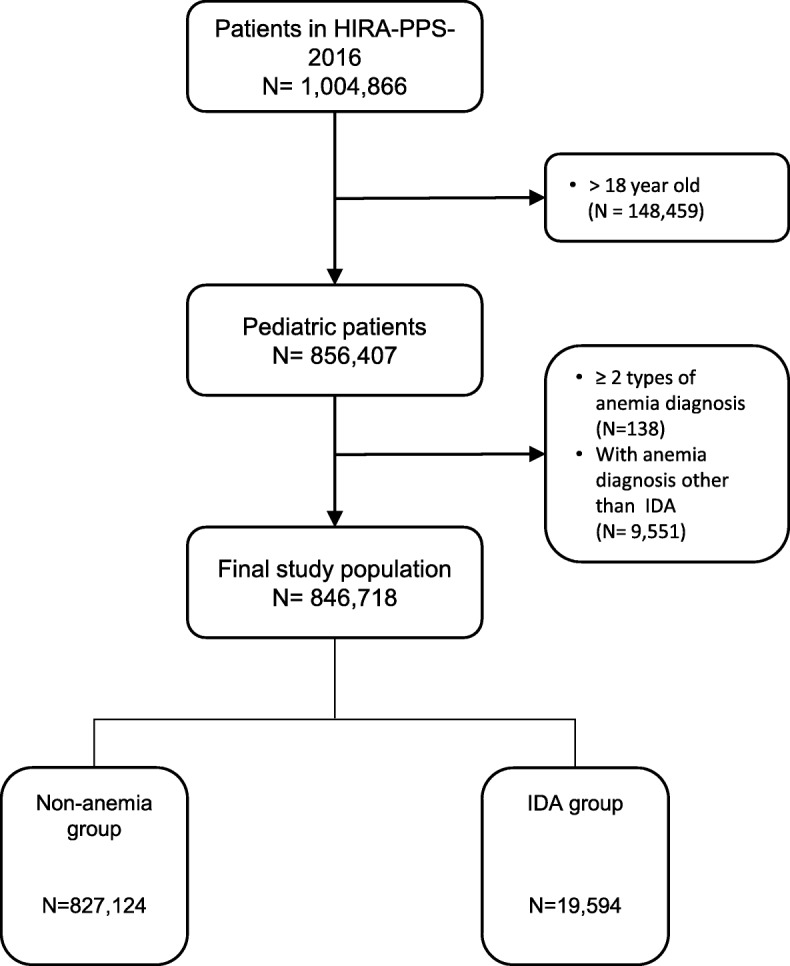
Flow diagram for study subject inclusion

Out of 1,004,866 patients included in the HIRA-PPS-2016, 148,459 patients were older than 18, 9551 were diagnosed with anemia other than IDA, and 138 were had more than one type of anemia diagnosis including IDA. Therefore, we included 846,718 pediatric patients (Fig. 1). The prevalence of all anemia was 3.46% (29,283 patients) in the subjects. The IDA group comprised 2.31% (19,594 patients). The OR of IDA was higher in patients younger [cOR = 0.89 (95% CI, 0.89–0.89); *P* < 0.001)], if age is as continuous variable, in female patients [cOR = 1.20 (95% CI, 1.17–1.24) relative to male patients; P < 0.001)], and in Medical aid patients [cOR = 1.20 (95% CI, 1.04–1.23; *P* = 0.006) relative to patients under health insurance program. All covariates, including systemic infection, mental disorder, CKD, chronic inflammation, cancer, and medication (steroid, methotrexate, and cyclosporine) indicated significantly higher ORs of IDA (Table 1).

**Table 1:** Patient characteristics according to iron deficiency anemia

	Frequency (%)		Crude OR (95% CI)	*P* value
	No anemia *N* = (827,124)	IDA (*N* = 19,594)		
Age	8.97 (5.16)^a^	5.98 (5.48)^a^	0.89 (0.89–0.89)	<.001
Sex				
Male	428,361 (51.79)	9253 (47.22)	1 [Reference]	
Female	398,763 (48.21)	10,341 (52.78)	1.20 (1.17–1.24)	<.001
Insurance Types				
Health insurance	806,892 (97.55)	19,054 (97.24)	1 [Reference]	
Medical aid	20,232 (2.45)	540 (2.76)	1.13 (1.04–1.23)	0.006
Systemic infection				
Meningitis	1645 (0.20)	130 (0.66)	3.35 (2.80–4.01)	<.001
BJI	803 (0.10)	41 (0.21)	2.16 (1.58–2.96)	<.001
Sepsis	3071 (0.37)	478 (2.44)	6.71 (6.09–7.40)	<.001
HEP	2445 (0.30)	695 (3.55)	12.40 (11.39–13.51)	<.001
CKD	57 (0.01)	26 (0.13)	19.28 (12.12–30.67)	<.001
Mental disorder				
Depression	3931 (0.48)	178 (0.91)	1.92 (1.65–2.23)	<.001
Anxiety	6518 (0.79)	309 (1.58)	2.02 (1.80–2.26)	<.001
Chronic inflammation				
PUD	15,248 (1.84)	640 (3.27)	1.80 (1.66–1.95)	<.001
COPD	12,650 (1.53)	422 (2.15)	1.42 (1.29–1.56)	<.001
SLE	228 (0.03)	26 (0.13)	4.83 (3.22–7.25)	<.001
RA	1454 (0.18)	96 (0.49)	2.80 (2.27–3.44)	<.001
IBD	265 (0.03)	68 (0.35)	10.87 (8.32–14.19)	<.001
Cancer	1147 (0.14)	133 (0.68)	4.92 (4.11–5.89)	<.001
Medication				
Steroid	333,265 (40.29)	11,726 (59.84)	2.21 (2.15–2.27)	<.001
Methotrexate	162 (0.02)	26 (0.13)	6.78 (4.48–10.27)	<.001
Cyclosporine	302 (0.04)	29 (0.15)	4.07 (2.78–5.96)	<.001

^a^mean (standard deviation)

*IDA* Iron deficiency anemia, *BJI* Bone and joint infection, *HEP* Hepatitis, *CKD* Chronic kidney disease, *PUD* Peptic ulcer disease, *COPD* Chronic obstructive pulmonary disease, *SLE* Systemic lupus erythematosus, *RA* Rheumatoid arthritis, *IBD* Irritable bowel disease

### Association of atopic disease and anemia

We found that the aOR of IDA was significantly higher across patients with atopic disease, at 1.42 (95% CI, 1.37–1.47; *P* < 0.001), 1.25 (95% CI, 1.21–1.29; P < 0.001), and 1.71 (95% CI, 1.65–1.76; P < 0.001) in patients with atopic dermatitis, allergic rhinitis, and asthma, respectively. All three atopic diseases demonstrated a significant, positive association with IDA before and after applying covariates (Table 2).

**Table 2:** Association between atopic diseases and iron deficiency anemia

Atopic diseases	Frequency (%)		Crude OR (95% CI)	*P* value	Adjusted OR (95% CI)	*P* value
	No anemia *N* = (827,124)	IDA (*N* = 19,594)				
Atopic dermatitis						
No	722,427 (87.34)	14,853 (75.80)	1 [Reference]		1 [Reference]	
Yes	104,697 (12.66)	4741 (24.20)	2.20 (2.13–2.28)	<.001	1.42 (1.37–1.47)	<.001
Allergic rhinitis						
No	534,731 (64.65)	10,344 (52.79)	1 [Reference]		1 [Reference]	
Yes	292,393 (35.35)	9250 (47.21)	1.64 (1.59–1.68)	<.001	1.25 (1.21–1.29)	<.001
Asthma						
No	600,218 (72.57)	9393 (47.94)	1 [Reference]		1 [Reference]	
Yes	226,906 (27.43)	10,201 (52.06)	2.87 (2.79–2.96)	<.001	1.71 (1.65–1.76)	<.001
Atopic diseases, No.						
0	388,310 (46.95)	5170 (26.39)	1 [Reference]		1 [Reference]	
1	280,108 (33.87)	6638 (33.88)	1.78 (1.72–1.85)	<.001	1.30 (1.25–1.35)	<.001
2	132,230 (15.99)	5804 (29.62)	3.30 (3.17–3.42)	<.001	1.81 (1.73–1.89)	<.001
3	26,476 (3.20)	1982 (10.12)	5.62 (5.33–5.93)	<.001	2.58 (2.43–2.73)	<.001

*IDA* Iron deficiency anemia

Adjusted by age, sex, insurance type, meningitis, bone and joint infection, sepsis, hepatitis, depression, anxiety, chronic kidney disease, peptic ulcer disease, chronic obstructive pulmonary disease, systemic lupus erythematosus, rheumatoid arthritis, irritable bowel disease, cancer, steroid, methotrexate, cyclosporine

Multiple atopic diseases in a single patient had higher associations with IDA than a single diagnosis. The aOR for 1 atopic disease and IDA was 1.30 (95% CI, 1.25–1.35; *P* < 0.001), increased to 1.81 (95% CI, 1.73–1.89); *P* < .001) in those with 2 atopic diseases, and was highest in patients with 3 atopic diseases [aOR = 2.58 (95% CI, 2.43–2.73; P < 0.001)] (Table 2). We also reported full multivariable logistic regression models of association between IDA and atopic disease in Additional file 1: Table S2. In addition, there was no evidence of multicollinearity among covariates. Maximum VIF (variance inflation factor) was lower than 2.0, and lowest eigenvalue was 0.32.

## Discussion

In this study, we showed that prevalence of IDA was associated with three atopic diseases (atopic dermatitis, allergic rhinitis, and asthma) by a large-scale data analysis of nearly 850,000 South Korean pediatric patients. We also found that the aOR of IDA was increased in children with multiple atopic disease diagnoses. Our results showed that comorbid medical conditions, the use of medication, gender, age, and socio-economic level were associated with the prevalence of IDA.

The prevalence of anemia in pediatric patients was approximately 3.42% in this study, the majority of which was IDA (2.31%); this figure is lower than the results of existing epidemiology studies, which suggest rates between 4 and 6% [12, 13]. The apparently low diagnosis of anemia in our study may be due to the use of the HIRA-PPS-2016 dataset because, in South Korea, most health screenings for infants and young children are brief physical examinations and do not routinely involve testing for anemia.

The etiology of anemia varies. AI is an anemia caused by inflammatory immune activation [14, 15]. AI produces similar hematological findings to iron deficiency anemia (IDA), including low hemoglobin and iron levels, and shows similar clinical manifestation to anemia in patients with chronic obstructive pulmonary disease, CKD, and congestive heart failure [16, 17]. We hypothesized that inflammatory conditions in patients with atopic diseases would increase their risk of anemia because previous studies have shown that inflammation, CKD, cancer, and systemic infection correlate with AI [18–22]. Indeed, in our study, the association of these diseases and medication was also high in patients diagnosed with IDA.

Inflammatory disease, malignancy, and infections are the major medical conditions underlying AI. In addition, combined anemia of IDA and AI is typically presented in patients with irritable bowel disease (IBD), or gastrointestinal (GI) malignancy [23]. Inflammatory diseases or depression would be a confounding factor which impact on both risk of anemia and atopic disease. Patients with moderate to severe atopic disease were treated by immunosuppressant such as methotrexate, cyclosporine, or steroids. Those medications could cause bleeding, anemia, or hematologic disorders [24–26]. Therefore, we included those medical condition and medication as covariates to adjust for minimizing their effects on outcome.

Most covariates we included were still associated with increased prevalence of IDA. Chronic obstructive pulmonary disease (COPD) and methotrexate, however, showed null effects after adjusting all covariates. As is well known, COPD is a disease that increases in incidence after middle age. We only included pediatric patients in this study, so it may have led to differences in outcomes from other inflammatory diseases in the outcome of COPD. Methotrexate is an antimetabolite and antifolate, which leads low level of folic acid in the body. Hematologic toxicity such as anemia in the methotrexate users would be more like folate deficiency anemia not IDA.

Because AI has a hematological manifestation similar to IDA [27], hepcidin is a useful indicator to differentiate AI from IDA [28, 29]. Unfortunately, hepcidin is rarely assessed in common clinical settings. Therefore, patients with AI may be diagnosed with IDA instead of AI, which would result in an artificially inflated risk of IDA in atopic disease patients.

In this study, we found that multiple atopic disease diagnoses in a patient had stronger associations with IDA, which is consistent with previous research [14]. Notably, Drury’s study showed allergic rhinitis was not associated with anemia. Our study indicated that the aOR of allergic rhinitis, although statistically significant, was lower than that for other atopic diseases (asthma and atopic dermatitis). The effect of systemic inflammation may therefore differ among atopic diseases.

We confirmed that the prevalence of IDA in pediatric patients with atopic dermatitis, allergic rhinitis, and/or asthma is higher than in patients without atopic disease. Because previous research has not provided evidence that atopic patients are at risk for anemia, monitoring and treatment guidelines for anemia in pediatric patients with atopic disease do not exist. However, treatment for anemia in children is crucial because of its potential to reduce quality of life [30], increase morbidity [31–34], and negatively affect school performance and physical growth [35, 36]. Therefore, we suggest that appropriate treatment of patients with atopic disease may require assessment for and treatment of IDA or AI.

Our study has several limitations. First, it is a cross-sectional study, and, thus, we cannot determine a causative relationship between atopic disease and anemia. However, the reverse causation—that anemia causes atopic disease—is unlikely given the pathophysiology of anemia and atopic disease. Second, because of its similarities to IDA, we have not directly assessed AI, nor have we identified whether the inflammatory state of atopic disease causes AI. Third, we cannot completely rule out selection bias because healthy children are more likely not to have screening for anemia. However, HIRA reported that most of the population use medical service at least once a year. In addition, unlike other serious health condition or medication use we included as covariates, anemia testing is not common in children with atopic disease, so the impact on outcomes would be limited. Nevertheless, this study has strengths, such as the inclusion of medical conditions and medications as covariates. We used diagnostic code, not self-report, for definition of disease. Lastly, by utilizing a large dataset sampled from national healthcare claims, we were able to objectively assess the association between atopic disease and anemia.

## Conclusions

We suggest that the inflammatory conditions that characterize atopic diseases (atopic dermatitis, allergic rhinitis, and asthma) could associated with IDA and/or AI. Further study is needed to clarify the distinction between IDA and AI to better understand the cause of anemia in patients with inflammatory diseases.

## Supplementary information

**Additional file 1: Table S1.** The list of KCD-7 diagnostic codes of diseases in the study. **Table S2.** Adjusted odds ratio of IDA for all covariates in final model.

## Data Availability

The datasets analysed during the current study are available from the corresponding author on reasonable request.

## Abbreviations

AI: Anemia of inflammation
aOR: Adjusted odds ratio
CI: Confidence intervals
CKD: Chronic kidney disease
COPD: Chronic obstructive pulmonary disease
cOR: Crude odds ratio
GI: Gastrointestinal
HIRA-PPS: Health Insurance Review and Assessment Service-Pediatric Patient Sample
IBD: Irritable bowel disease
ICD-10: International Classification of Diseases 10th Revision
IDA: Iron deficiency anemia
KCD-7: The Korean Standard Classification of Disease and Cause of Death-7
OR: Odds ratio
