# Supplementary material for: Association between atopic disease and anemia in pediatrics: a cross-sectional study
Source: BMC Pediatr. 2019 Nov 25;19:455. doi: 10.1186/s12887-019-1836-5 (PMC6876088; doi:10.1186/s12887-019-1836-5)
Supplement: Supplementary file 1 — Additional file 1: Table S1. The list of KCD-7 diagnostic codes of diseases in the study. Table S2. Adjusted odds ratio of IDA for all covariates in final model. [file 12887_2019_1836_MOESM1_ESM.docx]

**Additional file 1: Table S1. The list of KCD-7 diagnostic codes of diseases in the study.**

If the patients have diagnostic code of atopic disease once or more in a year, we defined the patients had the corresponding disease. Korean standard classification of disease and cause of death-7 (KCD-7) was used for definition of disease. KCD-7 reflects the update of the International Classification of Diseases (ICD-10), and refined the Korean subtype disease and rare disease to improve medical terms. Codes could be in any field of the encounter claim (primary or secondary diagnoses).

| **Category** | | **KCD-7** |
| --- | --- | --- |
| Atopic disease | Atopic dermatitis | L20 |
|  | Allergic rhinitis | J30.1, J30.2, J30.3 |
|  | Asthma | J45 |
| Anemia | Iron deficiency anemia | D50 |
| Covariates | Meningitis | A87, A39 |
|  | Bone and joint infection | M00, M01, M02, M03 |
|  | Sepsis | A40, A41 |
|  | hepatitis | B15, B16, B17, B18, B19 |
|  | Sepsis | A40, A41 |
|  | Chronic kidney disease | N18 |
|  | Depression | F32, F33 |
|  | Anxiety | F40, F41, F93, F06.4 |
|  | Peptic ulcer disease | K25, K26, K27 |
|  | Chronic obstructive pulmonary disease | J42, J43, J44 |
|  | Systemic lupus erythematosus | M32 |
|  | Rheumatoid arthritis | M05, M06, M08.0 |
|  | Irritable bowel disease | K51, K50 |
|  | Cancer | C** |

**Table S2. Adjusted odds ratio of IDA for all covariates in final models**

|  | | | adjusted OR (95% CI) | | | |
| --- | --- | --- | --- | --- | --- | --- |
|  |  |  | Atopic dermatitis | Allergic rhinitis | Asthma | No. of atopic diseases |
| Age | | | 0.89 (0.89-0.90) | 0.89 (0.89-0.89) | 0.90 (0.90-0.91) | 0.90 (0.90-0.91) |
| Sex | | |  |  |  |  |
|  | Male | | 1 [Reference] | 1 [Reference] | 1 [Reference] | 1 [Reference] |
|  | Female | | 1.23 (1.20-1.27) | 1.23 (1.20-1.27) | 1.25 (1.21-1.28) | 1.25 (1.21-1.28) |
| Insurance Types | | |  |  |  |  |
|  | Health insurance | | 1 [Reference] | 1 [Reference] | 1 [Reference] | 1 [Reference] |
|  | Medical aid | | 1.41 (1.29-1.54) | 1.43 (1.31-1.56) | 1.42 (1.30-1.55) | 1.42 (1.30-1.55) |
| Systemic infection | | |  |  |  |  |
|  | Meningitis | | 2.19 (1.81-2.64) | 2.15 (1.79-2.60) | 2.15 (1.78-2.59) | 2.13 (1.77-2.57) |
|  | BJI | | 2.25 (1.63-3.12) | 2.22 (1.60-3.07) | 2.27 (1.64-3.14) | 2.18 (1.57- 3.02) |
|  | Sepsis | | 3.12 (2.81-3.45) | 3.12 (2.81-3.46) | 3.10 (2.79-3.43) | 3.07 (2.77-3.41) |
|  | HEP | | 9.00 (8.21-9.86) | 9.03 (8.24-9.90) | 8.64 (7.88-9.47) | 8.71 (7.94-9.55) |
| CKD | | | 23.22 (13.77-39.13) | 23.32 (13.83-39.32) | 24.04 (14.26-40.51) | 24.05 (14.29-40.47) |
| Mental disorder | | |  |  |  |  |
|  | Depression | | 2.32 (1.96-2.74) | 2.31 (1.96-2.73) | 2.33 (1.97-2.75) | 2.32 (1.96-2.74) |
|  | Anxiety | | 2.17 (1.91-2.46) | 2.16 (1.90-2.46) | 2.12 (1.87-2.41) | 2.10 (1.85-2.39) |
| Chronic inflammation | | |  |  |  |  |
|  | PUD | | 2.40 (2.21-2.62) | 2.41 (2.22-2.63) | 2.37 (2.17-2.58) | 2.33 (2.14-2.54) |
|  | COPD | | 1.12 (1.01-1.24) | 1.10 (0.99-1.21) | 1.05 (0.95-1.16) | 1.03 (0.93-1.14) |
|  | SLE | | 1.96 (1.23-3.14) | 1.97 (1.23-3.15) | 1.98 (1.24-3.16) | 1.98 (1.24-3.16) |
|  | RA | | 2.74 (2.18-3.44) | 2.75 (2.19-3.46) | 2.73 (2.18-3.43) | 2.72 (2.16-3.41) |
|  | IBD | | 12.06 (9.01-16.12) | 11.98 (8.95-16.03) | 12.00 (8.95-16.07) | 12.04 (8.99-16.12) |
| Cancer | | | 4.04 (3.29-4.97) | 4.02 (3.27-4.95) | 4.08 (3.31-5.02) | 4.09 (3.32-5.03) |
| Medication | | |  |  |  |  |
|  | | Steroid | 1.80 (1.75-1.85) | 1.79 (1.73-1.84) | 1.63 (1.58-1.68) | 1.58 (1.53-1.63) |
|  |  | Methotrexate | 1.05 (0.64-1.70) | 1.06 (0.65-1.73) | 1.05 (0.65-1.71) | 1.14 (0.70-1.85) |
|  |  | Cyclosporine | 2.96 (1.95-4.50) | 3.52 (2.30-5.37) | 3.63 (2.38-5.55) | 3.15 (2.07-4.79) |

IDA: iron deficiency anemia, BJI: bone and joint infection, HEP: hepatitis, CKD: chronic kidney disease, PUD: peptic ulcer disease, COPD: chronic obstructive pulmonary disease, SLE: systemic lupus erythematosus, RA: rheumatoid arthritis, IBD: irritable bowel disease
